# The Possible Mechanistic Basis of Individual Susceptibility to Spike Protein Injury

**DOI:** 10.1155/av/7990876

**Published:** 2025-06-24

**Authors:** Matthew Halma, Paola Vottero, James Thorp, Tina Peers, Jack Tuszynski, Paul Marik

**Affiliations:** ^1^Independent Medical Alliance, Washington, District of Columbia, USA; ^2^Open Source Medicine Foundation OÜ, Tallinn, Estonia; ^3^Department of Physics, University of Alberta, Edmonton, Alberta, Canada; ^4^Division of Maternal and Prenatal Health, The Wellness Company, Boca Raton, Florida, USA; ^5^The Menopause Consultancy, Betchworth, Surrey, UK

## Abstract

Injury from spike protein, whether induced by COVID-19 infection or vaccination, constitutes a significant health concern for numerous individuals. Considerable heterogeneity exists in individual responses to both COVID-19 infection and vaccination, despite the latter being principally more controlled and consistent than the wide variety of infection circumstances. This review explores the possible mechanisms by which the spike protein contributes to cellular and systemic damage, highlighting the importance of understanding these processes for developing effective diagnostics and treatments.

## 1. Introduction

The issue of differential vulnerability to spike protein–related diseases is a vital question for understanding the basis of injury and developing general and individualized treatments for individuals. Addressing vaccine injury and long COVID, both of which are at least partially mediated by the spike protein, is crucial for future treatment strategies. Individual susceptibility to these conditions can inform the development of personalized therapeutic approaches. Adverse events after vaccination have been far more common with COVID-19 vaccines than with any other licensed vaccine [1]. Due to the pathological mechanisms of the vaccine-encoded spike protein, the potential for damage can exist at low levels for long periods of time, and those having received a vaccine can be in a “sword of Damocles” situation for years or even decades. Long-term impacts of COVID-19 vaccines were not studied in depth at the time of approval and cannot be definitively ruled out [2].

The extent of subclinical danger, as well as the increase in sudden and unexplained deaths, motivates the diagnosis of vaccine injury through biomarkers. Given its role as a major pathological agent driving vaccine injury, long COVID, and acute COVID-19 infection [3], the presence and concentration of the spike protein or its subunits in plasma serve as valuable biomarkers for assessing related diseases.

The present study is organized as follows: Section 2 provides an overview of the possible sources of vaccination response heterogeneity. The response to vaccines varies significantly due to individual genetic differences, administration factors, and the quality of the vaccine batches.  Section 3 explores the pharmacogenomics of spike protein interactions and their effect on individual susceptibility and response. System-specific pathologies are described in more detail, providing an overview of how genetic factors affect individual vulnerability to spike protein–induced diseases. Lastly, Section 4 gives a brief overview of diagnostic biomarkers and assays that could be useful to distinguish postvaccination syndromes from viral infection and inform the development of targeted therapeutic strategies.

## 2. Sources of Variation

Individual variation in vaccine response can be due to multiple factors, both owing to the recipient's genetic makeup and health status, as well as factors surrounding the administration itself. Some differences manifest quite obviously, such as the recipient having an anaphylactic reaction to polyethylene glycol (PEG), where the causality is well understood and the clinical presentation immediately apparent [4]. Other sources of variation in vaccine response are more investigational, having less accumulated evidence, and causality may be more difficult to show.

In addition to those individual health factors, there is some variation between vaccine administrations. While, in principle, the product should be consistent, there are challenges to delivering a consistent product to billions of people [5]. Furthermore, the AstraZeneca COVID-19 vaccine demonstrated process and product-related impurities [6]. The main factors implicated in the variability of vaccination responses are described below.

### 2.1. Factors Affecting Individual Susceptibility

#### 2.1.1. Administration

Differences in vaccine dosing can have a substantial impact on vaccine response. Significant variations were noted in the occurrence of adverse events across different vaccine batches [7]. Though there have not been large-scale studies on differences in administration, the practice of aspiration may possibly contribute to different vaccine responses [8–10].

A higher cumulative dose of mRNA is more likely to be associated with AEs, and the relationship is evident in both brand-to-brand comparisons (where the Moderna COVID-19 vaccine delivers a higher mRNA dose than the Pfizer BNT162b2) [11]. Additionally, one's risk of a vaccine adverse event will be expected to increase with the number of doses received.

#### 2.1.2. Vaccine Type

In the United States, several COVID-19 vaccines were authorized for emergency use or received full approval by the U.S. Food and Drug Administration (FDA) [12]. The most widely used vaccine was the Pfizer-BioNTech (Comirnaty) mRNA vaccine. Initially granted Emergency Use Authorization (EUA) in December 2020, it received full approval in August 2021 for individuals aged 16 and older and was eventually approved for ages as young as 6 months [12]. The vaccine required two doses, spaced 3 weeks apart, with booster doses available later. Pfizer-BioNTech became the most administered vaccine, accounting for approximately 60% of vaccinations in the United States [13] due to early distribution agreements [14] and its suitability for younger populations compared to the alternatives [15].

The Moderna (Spikevax) vaccine, also an mRNA-based vaccine, was another prominent option. It received its EUA in December 2020, around the same time as Pfizer [12], and full approval in January 2022 for those aged 18 and older [16]. Moderna's vaccine was approved for children 6 months and up, with a two-dose regimen spaced 4 weeks apart. Booster doses were also made available. Moderna vaccines accounted for about 37% of vaccinations.

Johnson & Johnson (Janssen) offered a viral vector vaccine that was initially popular due to its single-dose regimen. It received EUA in February 2021 for adults aged 18 and older [12]. However, concerns emerged over rare but serious side effects, such as blood clots, leading to reduced usage over time. Early on, J&J was a larger proportion of total doses but declined to 3% [12] as the J&J vaccine was discontinued owing to safety concerns [17].

Later in the vaccination campaign, the Novavax vaccine was introduced. It used a protein subunit technology and received EUA in July 2022 for adults aged 18 and older [18]. Novavax required two doses administered 3 weeks apart. Its uptake was relatively low, particularly since it was introduced after the majority of the population had already received other vaccines. Novavax accounted for less than 1% of the total vaccinations [13].

As the pandemic evolved, mRNA bivalent boosters from Pfizer and Moderna were introduced in 2022. These updated vaccines targeted both the original strain of the virus and the Omicron subvariants. By late 2022 and into 2023, these bivalent boosters became dominant in booster shot administration, as they were adapted for variants.

There are some structural differences between the spike depending on if one received an updated booster or one of the original vaccines.

#### 2.1.3. Contents and Purity

Concentrations of genetic material could vary, stemming from discrepancies in manufacturing quality and potential heterogeneity within each vial [19]. There are variances in the levels of adverse reactions, depending on the lot number of the vaccine [7, 20].

Another factor is the presence of adulterants, including DNA plasmids in the mRNA products [21]. It is known that Pfizer used a different manufacturing process for vaccine production than was used in the clinical trials, and the technique used in mass production may be more vulnerable to adulteration [22, 23].

### 2.2. Patient Characteristics

Viewing the series of events sequentially, the RNA must enter the cell and be read by the ribosome to express spike protein, after which it is degraded (in principle). Lacking an intact degradation pathway, the modified RNA will linger around and keep producing spike protein in principle. Minimal information is known about the biodegradation of modified mRNA in humans [2].

Vaccine response may depend on other factors as well. For example, time of day [24, 25], level of recent sleep [26–29], and prior infection [30] affect the immune response to COVID-19 vaccines. Several interventions have undergone clinical trials to determine their impact on the vaccine immune response, including iron supplementation (NCT04915820), mushroom mixtures (NCT04951336), immunomodulators (NCT04877496 [31] and NCT05060991), the ketogenic diet (NCT05163743), metformin (NCT03996538), probiotics (NCT05195151) and osteopathy (NCT04928456 and NCT05069636). Of these trials, only the trial on the immune impact of anti-CD20 therapy had results, which were not significantly different from controls [31].

#### 2.2.1. Variation in Cell Uptake and Biodistribution

The most crucial factor here is whether the injection was performed into the deltoid muscle, as intended, or some escaped into the bloodstream, which is largely set by the conditions of the injection itself (if aspiration was standard procedure). Individuals vary in their cellular affinity to take up extracellular RNA via endocytosis [32–34]. While it is unclear of their relevance in this case, there are genetic variations in endocytic pathways [35, 36].

Limited pharmacodynamic data existed on the novel use of LNP particles containing modified mRNA. The adenovirus and protein subunit vaccines had a more predictable pharmacodynamic profile, owing to previous data, which was limited for mRNA vaccines [2]. The biodistribution studies which did exist for mRNA-LNP products demonstrated distribution throughout the body [37], and later studies have found vaccine contents or artifacts in bodily fluids, including the breast milk of vaccinated mothers [38–40].

#### 2.2.2. Variation in Spike Protein and RNA Persistence

The existing literature on genetic determinants of COVID-19 vaccine adverse events is very limited [41]; therefore, we provide a mechanistic understanding. After endocytosis, the modified mRNA is present in the cytosol to be expressed, which can be highly variable depending on host genetics [42, 43]. From this point on, the major factors regulating the amount of pathogenic spike protein produced are the clearance and degradation mechanisms acting on the chemically modified mRNA. While pseudouridinylated RNA is far more stable than RNA [44], the degradation curve of N1-methyl pseudouridinylated RNA is less known, though N1-methyl-pseudouridine in mRNA has been observed to enhance translation [45]. Individual genetic variation influences the degradation of exogenous RNA [46].

Furthermore, other wildcards include the potential for reverse transcription into the host genome [47]. Another point of concern is the observed contamination of mRNA vaccine vials with the DNA plasmid vector [48], which may possibly be a factor in the observed persistence of spike protein in vaccine-injured patients [49]. This could be explained by transfection of bacteria in the human gut [50–52]. Were this a significant factor, we would expect differential vaccine injury susceptibility based on gut microbiota composition. Long COVID susceptibility varies according to gut barrier health [53], and acute SARS-CoV-2 infection severity is negatively associated with gut microbial diversity [54]. Long COVID is associated with higher levels of *Ruminococcus gnavus* and *Bacteroides vulgatus* and lower levels of *Faecalibacterium prausnitzii* [55]. Still, it is unknown to what degree this “gut reservoir” hypothesis for spike protein persistence is significant.

**Figure 1:**
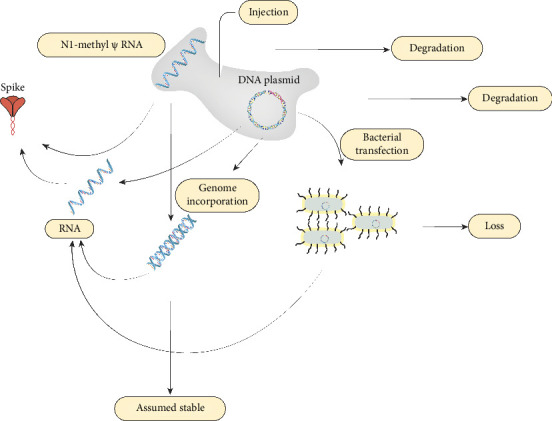
A model for long-term spike protein persistence in vaccine-injured individuals. This figure is an original figure created by the authors. A version of this figure appears in a preprint authored by the first author [56].

The primary factors are the variations in the initial dose of spike protein–encoding mRNA, which can vary due to storage, dilution, and administration. Once the mRNA is in the body, the level of spike results from the competition between mRNA degradation and protein expression from the mRNA, as shown in Figure 1.

We propose a third alternative mechanism to degradation and expression: conversion to a reservoir, as reverse transcription into the genome is possible [47]. Additionally, DNA contamination was discovered in a broad swathe of mRNA vaccine vials [48], potentially opening the possibility of gut microbiota transfection through the mechanism of horizontal gene transfer [57].

While the half-life of RNA is well known, and endogenous mRNA has a half-life of approximately 10 h [58], it is known that pseudouridinylated RNA is far more persistent [44, 59], and less is known about the degradation of the N1-methyl-psuedouridnylated RNA used in the mRNA vaccines [2]. The persistence of spike protein appears to be the differentiating factor between the presence or absence of postvaccination myocarditis [49]. Attempts have been made to quantify the lifetime of spike protein and mRNA in the human body [60], but it is unknown why some people appear to harbor the spike protein for longer durations than expected [61].

Tissue variation exists in the cellular uptake of modRNA containing LNPs, as factors such as LNP production processes [62] and individual genetic variation can impact delivery of modRNA to cells. Host factors impacting biodistribution and pharmacokinetics have been investigated for other drugs [63, 64], but the topic is not well understood for novel LNP-modRNA products. The closest examples are liposomal drugs, which are delivered with a liposome, often PEGylated [65], similar to the delivery vehicle of modRNA vaccines [66]. Individual variation in pharmacokinetic profile is more pronounced for liposomally encapsulated drugs than their nonencapsulated counterparts [65, 67].

Younger people tend to clear LNP-based drugs faster [65, 67, 68], but their rates of protein synthesis are also higher [69, 70]. Women also generally clear liposomal drugs slower than men [71], and this effect is possibly due to differences in body composition or organ differences in the liver or spleen [72].

External factors such as heat, light, or ultrasound may also play a role in LNP drug kinetics [66].

In principle, LNPs could be engineered to deliver to specific tissues [73, 74], but the Pfizer and Moderna modRNA vaccines showed high promiscuity, and entering into multiple tissue and organ types [37].

Differential capacity for protein synthesis exists between individuals due to many factors [75], including genetic [76]. These factors could affect the levels of spike protein produced following exposure to RNA vaccines or viral infections, potentially influencing the magnitude of tissue-specific responses. Although direct evidence linking tissue-specific protein synthesis efficiency to spike protein–induced adverse effects is currently lacking, this area warrants further investigation. Research focusing on the systemic biodistribution and tissue-specific localization of RNA and the resulting spike protein is crucial to better understand the interplay between protein synthesis and adverse outcomes following exposure.

In addition to RNA persistence and genomic considerations, SARS-CoV-2 spike protein mutations have implications for vaccine efficacy and disease severity. Notable mutations such as D614G, N501Y, and E484K are prevalent among variants of concern and significantly affect viral infectivity, transmissibility, and antibody evasion. For instance, D614G enhances infectivity by improving spike protein stability and ACE2 receptor affinity [77, 78], whereas N501Y similarly strengthens receptor binding, thereby increasing transmissibility [79]. The E484K mutation allows escape from neutralizing antibodies elicited by previous infection or vaccination, potentially reducing vaccine effectiveness [80]. These mutations highlight the necessity for ongoing surveillance and adaptive vaccine strategies.

#### 2.2.3. Pharmacogenomics of Spike Protein Interaction

Differential infection effects have been observed for different variants of SARS-CoV-2. Generally, later strains are milder than earlier strains, following a well-described evolutionary path for respiratory viruses [81]. Additionally, tissue tropism tends to move from the lower to the upper airways [81].

Each host has unique genetic makeup and unique environment, which mediate their interaction with (unique) viruses. The genetic factors that influence COVID-19 infection have been investigated through genome-wide association studies (GWAS), associating markers of disease severity with an individual's genetic makeup. One such study found that gene polymorphisms of proteins involved in coagulation were associated with acute COVID-19 prognosis [82]. These include MBL2 [83], ADAMTS13 [84], F8 [85], and PDGFRL [85], which are involved in clotting and have also been observed to be associated with acute COVID-19 severity. Our search reveals limited GWAS studies of long COVID [86, 87] or COVID-19 vaccine adverse events [41], though GWAS studies of SARS-CoV-2 vaccine antibody responses have been performed [88, 89] and several mechanistic factors behind the differential vaccine response are identified [90]. Genetic correlates of vaccine adverse reactions have been studied in the context of Hepatitis B, measles, rubella, and other vaccines [91].

Pharmacogenomics is another approach for determining the mechanistic basis of individual host susceptibility. While GWAS is observational, a pharmacogenetic approach can generate hypotheses for gene loci associated with differential vulnerability and their impact. For each gene that spike protein directly interacts with, changes in both spike and host receptor conformation will impact the interaction. While interactions are complex phenomena, broadly speaking, the energetic stability of the interaction is generally positively associated with the impact. This relationship holds for drug impacts on receptors [92].

In this analogy, the spike protein becomes the “drug”, and its impact will depend on the sequence and subsequent conformation of the human receptors that interact with it. Furthermore, as the spike protein interacts with multiple proteins, the interaction with host genetics is more complex and multifactorial.

**Figure 2:**
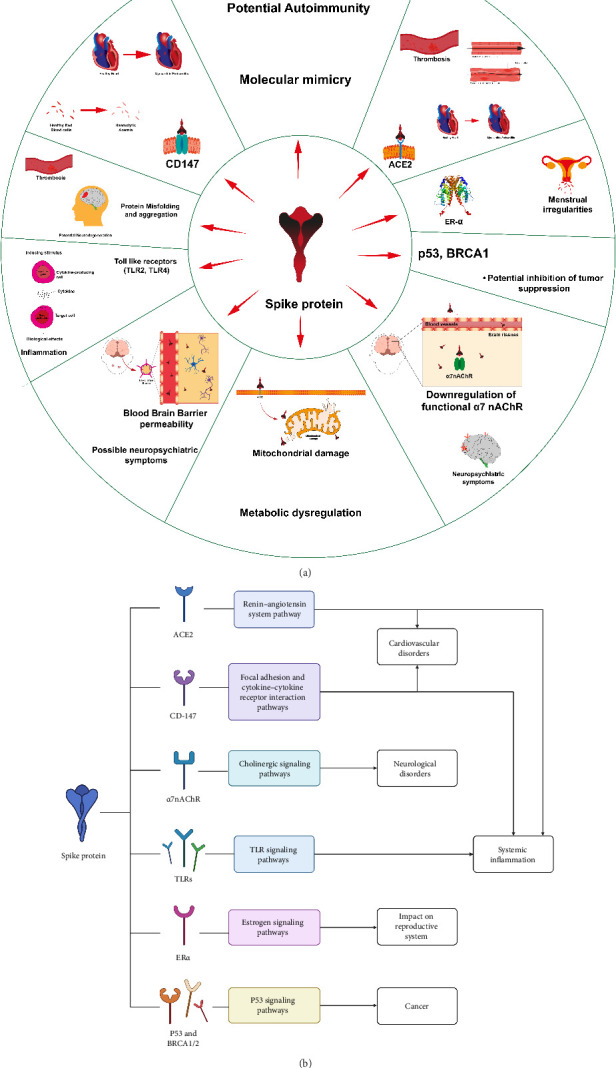
Pathophysiology related to spike protein. (a) Pathophysiological interactions of the spike protein. Slices show potential mechanisms (inside) and subsequent pathophysiology (outside). This figure has been reproduced from [95] under the permission of CC Attribution 4.0 international license (CC BY 4.0 DEED, https://creativecommons.org/licenses/by/4.0/). (b) Pathways and associated pathology. This figure panel is an original production by the authors.

Several recent reviews have provided comprehensive pictures of the pathological mechanisms of spike protein [93–97]. The pathological impacts on different organ systems are described in Figure 2 and Table 1 [95].

The following section covers system-specific pathologies. Although some interaction pairs contribute to pathologies across multiple systems, we will discuss the first instance of the pharmacogenetic basis of variation.

## 3. System-Specific Pathologies

### 3.1. Cardiovascular Disorders: ACE2, CD-147, and Anti-PF4 Antibodies

Cardiovascular disorders were the first vaccine-related safety concern to be observed [145] and acted upon [146]. Several other countries discontinued the AstraZeneca vaccine in the wake of cases of myocarditis, especially in young males [146]. Later, Moderna vaccines were restricted in young people in some European countries [147].

While controversy exists over the rates of cardiovascular disease following COVID-19 vaccination, a survey of Thai boys aged 13–18 receiving a Pfizer/BioNTech BNT162b2 revealed that 2.3% of the boys had at least one elevated cardiac biomarker or positive lab assessment, and 29% had at least one cardiac manifestation, such as tachycardia, palpitation, or myopericarditis [148]. Markers of inflammation as well as clotting were elevated in groups receiving either mRNA or AstraZeneca vaccines compared to controls [118]. Spike protein demonstrably damages human cardiac pericytes independent of infection [106]. It should be noted that variation in disease susceptibility is not restricted to the below mechanisms, for example, coagulopathies are implicated in some cases of vaccine injury [149] and long COVID [150]; therefore, genetic factors underlying coagulability may be associated with the prognosis of either condition.

#### 3.1.1. ACE-2

ACE-2 is the most prominent interaction partner for spike protein, as it is the primary mechanism by which cell entry is mediated [151]. Variations in ACE2 conformation owing to genetic variation may result in differential susceptibility to COVID-19 infection and variation in disease course and severity [152].

Interactions with ACE-2 can have metabolic consequences [153], as well as consequences for hypertension [151]. Long-term stimulation of the receptors can result in ACE-2 downregulation, which can lead to metabolic abnormalities [153].

Several compounds may disrupt the binding interface between spike protein and ACE2, notably ivermectin [154], quercetin [155], and *N*-acetyl cysteine [156].

**Figure 3:**
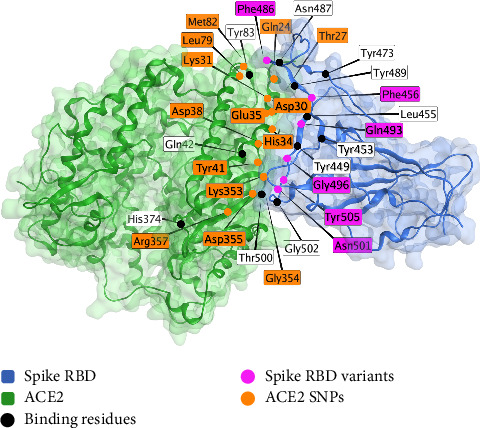
The interaction interface between the spike protein receptor binding domain (RBD, blue) and the angiotensin-converting enzyme 2 (ACE-2) receptor (green) (PDB ID: 6LZG, X-ray diffraction, resolution 2.5 Å) [168]. Interacting residues are shown as black dots, and variable residues with SNPs are shown in violet for variation in the spike protein and orange for variation on the ACE2 receptor. This figure is an original figure created by the authors.

The pharmacogenomics of the ACE2 receptor is the best-studied interaction out of any human receptor interaction with spike protein. Several sites of human host variation are documented to be associated with COVID-19 disease outcomes. These include I21V, E23K, K26R, T27A, N64K, T92I, Q102P, and H378R [157], Arg514Gly [158], rs2106806 AND rs2106807 in combination [159], rs6629110 [159], K31R and E37K (lowered susceptibility), K26R and T92I (raised susceptibility) [160, 161], S19P, I21V, E23K, K26R, T27A, N64K, T92I, Q102P, and H378R (raised susceptibility), K31R, N33I, H34R, E35K, E37K, D38V, Y50F, N51S, M62V, K68E, F72V, Y83H, G326E, G352V, D355N, Q388L, and D509Y (lowered susceptibility) [161], rs35803318/Val749Val and rs4646179/Asn690Asn [162], K26R and S331F [163], Arg652 (interaction with TMPRSS2) [163], rs41303171 [164], rs35803318, rs41303171, rs774469453, rs773676270, and rs2285666 [165], rs4646120 and rs2285666 [166], and rs2074192 [167].  Figure 3 shows the sites of these variations in the spike receptor binding domain (RBD)–ACE2 complex.

A model of the spike protein–ACE2 interaction is shown below [168] (Figure 3).

#### 3.1.2. CD-147

Transmembrane glycoprotein CD-147 is an alternate mechanism by which the SARS-CoV-2 virus can enter cells [105]. Activation of CD-147 by spike protein has been implicated in some of the cardiovascular symptoms observed in long COVID and postvaccination syndrome patients [106, 169, 170]. While most therapeutic modalities ignore this interface, azithromycin is a potential downregulator of CD-147 [171].

**Figure 4:**
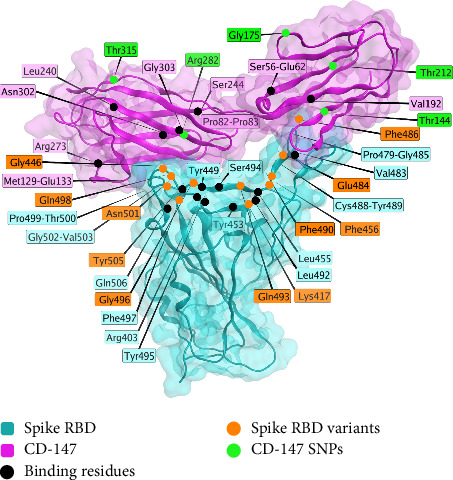
The interaction interface between the spike protein receptor binding domain (RBD, blue) and the CD-147 receptor (violet). The structural model represented here has been reproduced from the in silico model by Helal et al. [172], with permission from the authors. Variable residues on spike RBD are shown in orange, whereas variable residues on CD-147 are shown in green. Residues observed to interact are highlighted in black. This figure is an original figure created by the authors.

The interaction of spike protein and CD-147 is characterized structurally [172] interaction, and several associations have been observed [173]. CD-147 is an alternative entry route for the SARS-CoV-2 virus [168]. Models exist of the interaction between the two proteins (Spike and CD-147). Structurally, the RBD of spike protein interacts with several residues of the CD-147 (alternate name TMPRSS2) protein, as shown in Figure 4. SNPs of interest for interactions with spike protein include Rs8259T > A [174], rs371073966 [175], rs104894669 (E208K), rs2283574, rs6757, rs8637, rs4919862, rs6758, rs8259, rs4919859, and rs28915400 [176], and rs2283574, rs6757, rs8637, rs4919862, rs6758, rs8259, rs4919859, and rs28915400 [176]. An additional study identified differences in the noncoding region of CD-147 between vaccinated controls and infected individuals, albeit using a small sample size (*n* = 12) [177].

### 3.2. Anti-PF4 Antibodies

While it is controversial if the development of anti-PF4 antibodies is due to the spike protein, or another vaccine product, these have been observed in those experiencing vaccine-induced thrombosis and thrombocytopenia (VITT) [178, 179]. The development of anti-PF4 antibodies is thought to be related to the polyanion content of the adenovirus vector vaccines, both AstraZeneca and Johnson and Johnson, and not the spike protein.

### 3.3. Neurological Disorders: ACE2, Nicotinic Adrenergic Receptor (ADR), Protein Misfolding, Barrier Function, and Others

Neurological disorders have been observed after vaccination and also as part of the symptoms of long COVID. A review of case series and case reports found the most common serious CNS manifestations to be cerebral venous sinus thrombosis (CVST), mostly occurring after the first AstraZeneca dose (93% of total reports) [180]. Other frequently reported symptoms were CNS demyelinating disorders and encephalopathy/encephalitis. The most common manifestations in the peripheral nervous system were Guillain–Barre syndrome (GBS) and Bell's palsy (BP).

Other complications impacting the brain include intracerebral hemorrhages, strokes, akathisia (inability to remain still), delirium, seizures, and epilepsy [181]. The disorders transverse myelitis (TM), multiple sclerosis (MS), and neuromyelitis optica (NMO), an autoimmune disorder attacking the nervous tissue of the eyes and spinal cord, have also been documented after vaccination [181]. Other nervous system disorders include optic neuritis, anosmia (inability or reduced ability to smell), and tinnitus (unabating ringing sensation in ears). COVID-19 vaccination can, in some cases, reactivate Herpes Zoster, which can impair peripheral nervous sensation. Parsonage-Turner syndrome, characterized by shoulder weakness, was also reported after COVID-19 vaccination [181]. COVID-19 vaccines, as well as viral infection, can also result in postural orthostatic tachycardia syndrome (POTS) [182].

Less serious and more transient symptoms are very common after vaccination. In a survey of vaccine recipients experiencing neurological symptoms, 46% experienced fatigue, 36% experienced cognitive impairment, 30% experienced a headache, 10% experienced tinnitus, and 16% experienced vertigo [183]. In total, 32% of those with some neurological symptoms experienced some central nervous system manifestation and 40% experienced some peripheral nervous system manifestation. A survey of vaccinated individuals may provide more accurate insights into occurrence rates. 36% of vaccinees reported some long-term issue, 20% experienced myalgia, 14% experienced fatigue, 1% experienced paresthesia, 1% experienced ageusia, 2% experienced sadness or irritability, and 3% experienced lack of concentration or excessive worry [184, 185].

Cognitive impairment characterizes a subset of vaccine recipients [130, 134, 186–188]. Probable mechanisms include modulation of the ACE2 and nicotinic ADRs, protein misfolding, damage to blood–brain barrier (BBB) function, and molecular mimicry [95, 189]. Spike protein reduces burst activities in neurons, which may contribute to neurological symptoms [190].

It bears mentioning that the metabolic and cardiovascular effects of the spike protein also significantly impact nervous system disorders [95], and symptoms are not neatly separable by body system.

#### 3.3.1. ACE2

ACE2 interactions with spike protein may also contribute to neurological disorders [191, 192]. ACE2 is expressed in human neurons, and SARS-CoV-2 shows neurovirulence [193]. Activation of the Renin Angiotensin system, of which ACE2 belongs to, may promote inflammation, and are associated with cell death and cognitive impairment in the nervous system [194].

Spike protein is expressed during both COVID-19 infection and COVID-19 vaccination, and ACE2-mediated symptoms may be common to both [195].

#### 3.3.2. Protein Misfolding

Misfolding of SARS-CoV-2 spike protein and its translation fragments has been hypothesized as a mechanism contributing to neurodegenerative diseases [196]. Furthermore, laboratory data has observed aggregates formed when spike protein is seeded [196], and spike protein may potentiate the aggregation of other proteins [197]. Fragments of spike protein may also contribute to prion-like propagation of aggregates [198].

Generally, prion-like diseases are progressive [199]. However, aggrephagy, an autophagic process that consumes aggregates, is a possible therapeutic mechanism for the removal of aggregates [200].

The aggregation proneness of spike protein is not the only concern, as the use of N1-methyl-pseudouridine as a replacement for vaccine mRNA can also contribute to unintended ribosomal frameshifting [201], which may increase the aggregation proneness of translated fragments. Differences in translation were at the threshold of statistical significance in the initial experiments testing the translation fidelity of N1-methyl-pseudouridine [202].

#### 3.3.3. Functional α7 Nicotinic Acetylcholine Receptor (α7nAChR)

**Figure 5:**
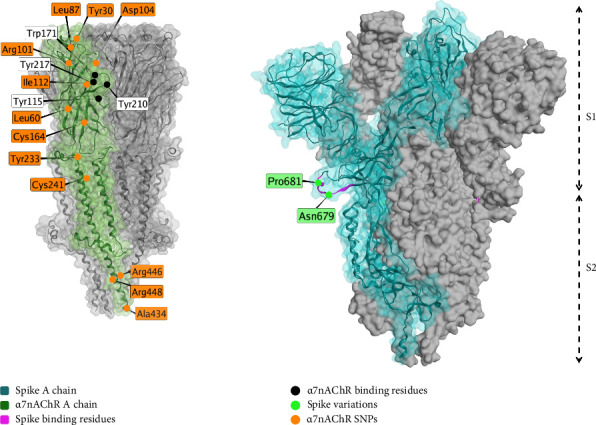
The interaction between spike (S) protein (blue, PDB ID: 7OAN [203], electron microscopy, resolution 3.0 Å) and functional α7 nicotinic acetylcholine receptor (α7nAChR, green, PDB ID: 7EKI [204], electron microscopy, resolution 3.18 Å). Interacting residues on α7nAChR are shown in black, where variable residues are shown in orange. Variable residues of the spike protein are shown in green. This figure is an original figure created by the authors.

The proposed interaction of spike protein with the α7nAChR, shown in Figure 5, is a potential mechanisms behind the cognitive and neuropsychiatric symptoms observed in individuals with long COVID or postvaccine syndrome [205]. α7nAChRs downregulate the production of proinflammatory cytokines [206], so interference can dysregulate inflammation. α7nAChRs play important roles in memory and appear in decreased numbers in conditions of memory disorders or neuroinflammation [207]. Prior studies show that electrical vagal nerve stimulation inhibits tumor necrosis factor (TNF) synthesis, a proinflammatory agent, and that this is mediated through α7nAChRs [206].

Spike protein was shown to dampen the electronic activation of other nicotinic cholinergic receptors, but did not affect α7nAChR [208].

In an acid-induced acute lung injury mouse model (unrelated to COVID-19), α7nAChR agonists, including nicotine, decrease inflammatory manifestations [209]. Agonists may be a potential therapeutic for the treatment of COVID-19 and long COVID [208], and the approach may possibly be broadened to other respiratory diseases [210].

The endogenous metabolite of nicotine, 1-methylnicotinamide (1-MNA), is shown to reduce postexercise fatigue in patients recovering from SARS-CoV-2 [211].

Beyond the direct interaction of spike protein with the α7nAChR receptor, α7nAChR also interacts with ACE2, and there may be a role for anti-idiotypic antibodies in long-term cognitive dysfunction following long COVID or SARS-CoV-2 vaccination [205].

#### 3.3.4. BBB Permeability

Spike protein has been observed not only to pass through the BBB [137] but also to induce permeability of the BBB to other proteins and to degrade barrier function [212]. Lack of BBB integrity is associated with cognitive issues [213, 214] and is proposed as a contributing factor to some neurological conditions [213]. COVID-19 demonstrates neurovirulence [215]. Vaccinal lipid nanoparticles may enter brain tissue [216], as spike protein of possible vaccine origin has been observed in brain tissue from postvaccine autopsies [217].

Several investigational therapeutics [218, 219] and nutraceuticals [220] have been explored for the restoration of BBB function, and may be of therapeutic benefit for neurological symptoms after vaccination [140] or COVID-19 infection [221].

### 3.4. Systemic Inflammation: Toll-Like Receptors (TLRs) and Autoantibodies

Systemic inflammation has been an observed adverse event of COVID-19 vaccination [118, 222], including immune thrombocytopenia [118], Still's disease [223], rheumatic diseases [224], and multisystem inflammatory syndromes (MIS) [222, 225–228].

Mast cell activation syndrome (MCAS) is a potential contributor to long COVID-like symptoms, as mast cells (MCs) are activated by SARS-CoV-2 [229]. People experiencing long COVID show a similar symptom profile to MCAS patients [229–231]. MCAS patients can present with general inflammation patterns in any system, including neuroinflammation.

Recent research has shown that SARS-CoV-2, including its spike protein, can modulate host immune pathways and may have the capacity to disrupt normal epigenetic regulation. For example, viral proteins can mimic histone structures, interfering with chromatin organization and gene transcription [232]. Additionally, infection-induced inflammatory signaling, particularly via TLR activation, can alter the activity of epigenetic enzymes, leading to changes in DNA methylation and histone modifications that affect immune gene expression and may contribute to persistent immune dysregulation [233]. Recent epigenetic immune monitoring studies suggest that such changes are relevant not only to the acute immune response but also to the disease course and prognosis in COVID-19 [234]. These epigenetic changes have been implicated in long-term health consequences and may partially explain individual variation in responses to infection or vaccination.

#### 3.4.1. TLR Activation

Stimulation of TLRs by spike protein has been observed in cell culture experiments and can contribute to an inflammatory cascade [235]. TLR activation may contribute to MCAS [236]. Much of COVID-19's hyperinflammation is concordant with manners of inflammation which MC activation can drive [237].

Antagonists of TLR4 may lower the inflammation associated with COVID-19, including sparstolonin B, which was studied prior to COVID-19 [238].

**Figure 6:**
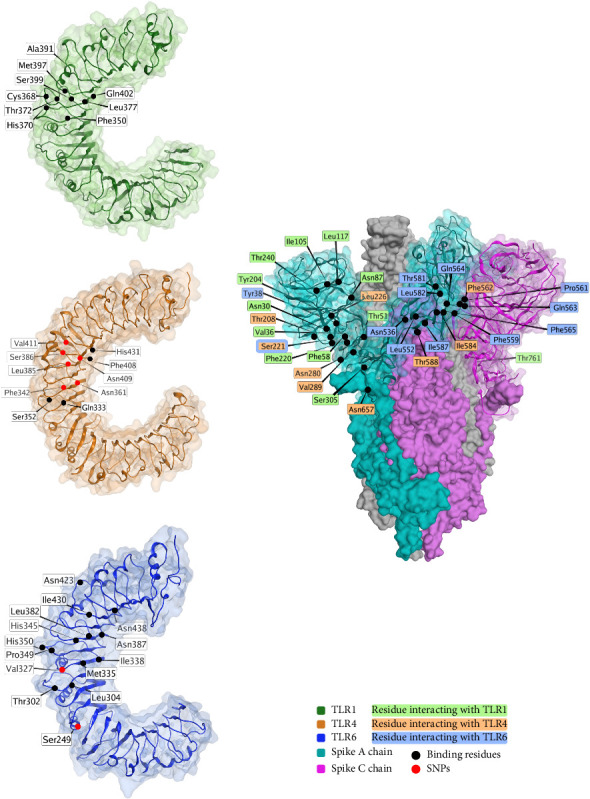
Interaction of spike protein with human toll-like receptors (TLRs) and the spike (S) protein (PDB ID: 7OAN [203], electron microscopy, resolution 3.0 Å). TLR1 (PDB ID: 6NIH [240], X-ray diffraction, resolution 2.3 Å) is shaded in green, TLR4 (PDB ID: 3FXI [241], X-ray diffraction, resolution 3.1 Å) is shaded in brown, and TLR6 (PDB ID: 3A79 [242], X-ray diffraction, resolution 2.9 Å) is shaded in blue. Binding residues are shown in black, and variable residues shown in red. Spike protein residues interacting with TLR1 are shaded in green, residues interacting with TLR4 are shaded in orange, and residues interacting with TLR6 are shaded in blue. This figure is an original figure created by the authors.

TLRs are a class of several proteins that are important for immune response, especially in response to foreign RNA. The spike protein is predicted to interact with several of the TLR proteins, and 3D models of the interaction exist for TLR1, TLR4, and TLR6 [239], as shown in Figure 6.

Several TLR polymorphisms are associated with COVID-19 outcomes, including in TLR1 (rs5743551 [243]), TLR2 (rs5743708 [244] and rs3804100 [243]), TLR3 (rs3775290 [245]), TLR4 (rs4986791 [244] and rs4986790 [246]), and TLR7 (rs179008 [245]).

#### 3.4.2. Molecular Mimicry

Molecular mimicry is a possible explanation for the anti-PF4 cascade observed in VITT patients. The production of autoantibodies is more often linked to specific rather than systemic inflammation. Several epitopes of the spike protein share conformational similarities to many human protein motifs, and autoantibodies may form, potentially triggering autoimmune attacks.

The spike protein possesses a TQLPP motif similar to thrombopoietin and an ELDKY, similar to the human protein PRKG1, involved in platelet activation [144].

Autoantibodies for ADR B1 and B2 and the CNS and vasoregulation-associated muscarinic acetylcholine receptor (CHR) M3 and M4 were associated with long COVID severity [247, 248]. Other relevant autoantibodies for ACE2, MDA5, CD255, SS-B/La, and PM/Scl-75 [249] exist, as well as autoantibodies for immune proteins [250]. Computational alignment to evaluate molecular mimicry of these antigens suggests plausibility. These proteins are highly variable in humans, and this may play a role in differential disease outcomes [251].

Other proteins with potential cross-immunity with spike protein are CHL1, ENTPD1, MEAF6, SLC35G2, and ZFHX2 [252].

### 3.5. Reproductive System: Estrogen Receptor Alpha

The spike protein may interact with high affinity with estrogen receptor alpha, which can result in menstrual abnormalities, as the delicate coordination of estrogen and other hormones regulates menstruation. Beyond menstrual cycle abnormalities [121, 253–269], other reproductive issues have been observed in vaccinated patients, including heavy menstrual bleeding [270, 271].

Studies on changes in the menstrual cycle document changes in roughly 40% of menstruating women receiving the first dose of a COVID-19 vaccine [121, 253–269]. Heavy menstrual bleeding occurred at higher rates in reproductive-aged women receiving a COVID-19 vaccine compared to this same group measured prior to vaccination, affecting 34.9% of vaccinated women of childbearing age within 28 days of their first dose, as opposed to 20.6% in an unvaccinated control group [272].

Thorp et al. using data from a US pharmacovigilance database known as the vaccine adverse event reporting system (VAERS), compared miscarriage rates of COVID-19 vaccines with those of other vaccines, finding significantly higher rates of miscarriage from COVID-19 vaccines compared to influenza vaccines [273].

**Figure 7:**
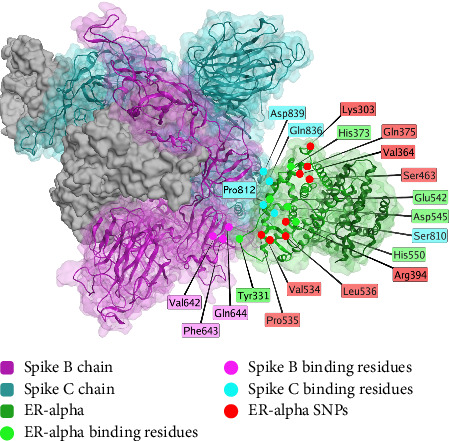
Interaction between spike protein (purple shading for Chain B and cyan shading for Chain C) and the estrogen receptor alpha (ERα) protein (green). Binding residues are shown in green for residues of ERα, and violet and cyan for residues of spike protein chains B and C, respectively. SNPs of ERα are highlighted in red. The structural model represented here has been reproduced from the model by Solis et al. [119], with permission from the authors. This figure is an original figure created by the authors.

While the precise mechanism for COVID-19 vaccine-induced menstrual changes has not been confirmed, one plausible explanation is that the spike protein interacts with the estrogen receptor alpha [119]. Structurally, the nuclear receptor coregulator (NRC) LXD-like motif on the S2 subunit [119] of the spike protein interacts with Helix 11 of the estrogen receptor, as shown in Figure 7.

The region of the spike protein interacting with ERα does not show a significant difference between SARS-CoV-2 variants (Table 2). Human host variation does exist in this region, but no studies have identified any correlation between genetic variants and menstrual outcomes (Table 2). The genetic variability in this region may be a possible explanation for differential reproductive system responses to COVID-19 vaccination.

Little is known about treating spike-related menstrual disturbances specifically. However, one word of caution should be noted: nattokinase [304] and bromelain should not be used in pregnancy and breastfeeding mothers because of the potential risk of bleeding complications in the preborn, newborn, and breastfed infants until there are more reassuring data available [305].

### 3.6. Cancer: P53, BRCA1/2, and Possible Genomic Instability

Much of the evidence for a carcinogenic role of COVID-19 vaccines in cancer remains anecdotal [306]. Limited case reports exist for the formation of neoplasms after vaccination, both malignant [307] and benign [308, 309]. During clinical trials, two cases of emergent malignancies occurred soon after vaccination, which may well be coincidental given the size of the trials [310].

Cases of cancer recurrence possibly attributable to COVID-19 vaccination have been reviewed recently [311]. Disease recurrence has been observed in select case reports [312, 313], as well as suspicious but ultimately benign recurrences [314–316]. In rare cases, vaccination can increase the risk of metastasis from lymph nodes [317].

#### 3.6.1. P53 BP1

The spike protein is computationally predicted to interact with the P53 and BRCA1 tumor suppressor proteins, which could be a possible contributor to carcinogenesis [122]. In addition, spike protein DNA inhibits the p53-mediated activation of proteins p21, TRAIL death receptor and MDM2, which increases cancer cell viability after chemotherapy [318].

#### 3.6.2. BRCA1/2

**Figure 8:**
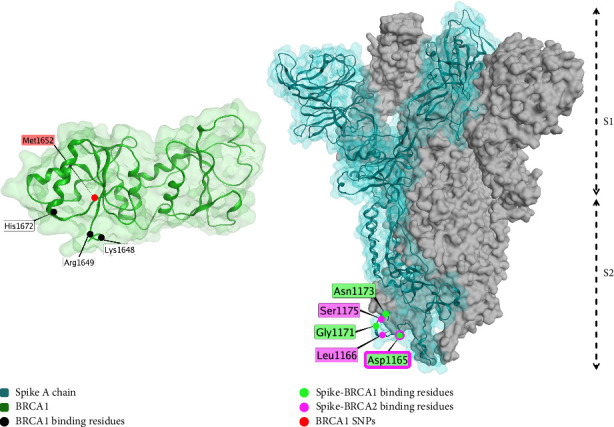
Interaction between spike protein (blue and gray, PDB ID: 7OAN [203], electron microscopy, resolution 3.0 Å) and BRCA1 (green, PDB ID: 3PXB [319], X-ray diffraction, resolution 2.5 Å). Binding residues of BRCA1 (interacting with spike protein) are highlighted in black. SNPs of BRCA1 are shown in red. BRCA1- and BRCA2-interacting residues on spike protein are highlighted green or violet, respectively. Asp1165 interacts with both BRCA1 and BRCA2. This figure is an original figure created by the authors.

The spike protein is predicted to interact with BRCA1 and 2 (Figure 8), which have tumor suppressor roles [122].

There are several possible sites for pharmacogenomic variation in patient response to vaccination; these are summarized in Table 2.

## 4. Diagnostics

Given that disease etiology is often attributed to the spike protein, the presence and concentration of the spike protein are suitable biomarkers for spike protein diseases. Additional biomarkers can probe other issues related to the spike protein, including inflammatory markers, markers of heart damage, and markers for blood coagulability.

Still, most diagnoses are made based on patient history and clinical presentation. While several spike protein assays exist, they are still mostly restricted to the research settings [320–322] and are not available as diagnostic tools. In the future, ideally, spike protein concentration assays will guide treatment, allowing for tracking progress as well as exploring other non-spike-related disease etiologies. If the pathology is spike protein–related, developing a diagnostic test may be a helpful step in assessing treatment progress.

The World Health Organization (WHO) guidelines for Adverse Events Following Immunization (AEFI) are widely used for causality assessment to evaluate the likelihood of a causal relationship between a vaccine and an adverse event. However, as highlighted by previously published critiques, the WHO AEFI guidelines have limitations that may hinder their ability to effectively identify causal relationships in the context of COVID-19 vaccines [323, 324].

The WHO AEFI causality assessment framework is designed primarily to identify clear, well-defined adverse events that follow vaccination. It requires that the type of adverse event be identified in large-scale epidemiological studies beforehand, placing a significant burden on when assigning causality [323]. As a result, rare, delayed, or complex adverse events—particularly those that may manifest differently across individuals due to genetic, age-related, or tissue-specific factors—are often excluded from causal determination.

Moreover, the guidelines are generally stringent about excluding coincidental events or those lacking strong immediate evidence of causality. Under the current guidelines, for causality to be demonstrated, a rechallenge with the agent must be performed to see if the symptoms manifest. This is not possible in the cases where the subject has died, and is not advisable in cases when the adverse event is life threatening. A paradox exists where the end result is, under the current criteria, it becomes virtually impossible for a vaccine ever to be considered a cause of death under the WHO AEFI criteria [323]. Deaths observed after vaccination are not considered as causally related to the vaccine unless there was a statistically significant increase in deaths during the trials with few participants that preceded approval. Such a vaccine would not be approved, which means that for vaccines and death, there is not functioning postmarketing surveillance [323].

### 4.1. General Diagnostics

In guiding treatment, there are multiple biomarkers that one can test to gain insight into the progression of the injury sustained from the vaccine. General biomarkers for cardiovascular risk, nonspecific for vaccine injury, include troponin, D-dimer, and C-reactive protein [49].  Table 3 summarizes the biomarkers for general diagnostics. It must be noted that these biomarkers are specific to cardiac injury and cannot be used to determine disease etiology.

Troponin is a general biomarker associated with diagnosis of acute coronary syndromes [327, 328], as troponins are released into the blood following damage to cardiac muscle [329]. D-dimer is a biomarker associated with the breakdown of fibrin clots by the fibrinolytic system [330]. As the test measures the breakdown of clots, a high measure can indicate a high level of clot burden and a high degree of breakdown [331], and this must be taken into consideration by the clinician.

C-reactive protein is an inflammatory biomarker, and higher values are associated with increased cardiovascular risk [49, 325, 326, 332].

### 4.2. Specific Diagnostics

A recent paper by Yonker et al. surveyed the biomarkers of vaccinated individuals, both with and without postvaccination myocarditis. The main differentiator between the group with myocarditis and those without was the persistence of full-length spike protein, unbound by antibodies [49]. Given that this is the sole gene encoded by most vaccines and has multiple documented pathological mechanisms [3], it is a likely etiological factor in postvaccination syndrome. Differentiation between spike protein of viral or vaccine origin can in principle be accomplished through several ways (Table 4).

Cases of blood thrombosis after vaccination typically occur within 1 month of receiving the injection [339, 340]. A test for spike protein contains two important quantities: 1) the concentration of spike protein and 2) the time since vaccination. Most often, the spike protein concentration drops off quickly within 1 week of the injection [341]. However, the persistence of high levels of spike protein for months after injection has been documented in a subset of vaccinated individuals [342]. While the specific factors affecting long-term spike protein levels are still unclear, we proposed a model for the long-term persistence of spike protein (detailed in Section 2.2.2).

In interpreting the causes of interpersonal variation in vaccine response, we restricted our analysis to disease etiologies tracing to the spike protein in the vaccines. While there may be other contributing factors, they are outside the scope of this review.

## 5. Conclusions

Addressing vaccine injury and long COVID, which are at least partially mediated by the spike protein, is crucial for future treatment strategies. Understanding the mechanisms of individual susceptibility to these conditions can guide the development of personalized therapeutic strategies.

We reviewed the possible sources of vaccination response heterogeneity, including administration and batch quality of the vaccines and individual genetic differences in the population. We provided a detailed exploration of the pharmacogenomics of spike protein interactions, giving a mechanistic explanation of how genetic factors affect individual vulnerability to spike protein–induced, system-specific diseases. We reviewed diagnostic biomarkers and assays that could be useful to track cardiovascular damage, distinguish postvaccination syndromes from viral infection, and inform the development of targeted therapeutic strategies. While current diagnostic methods primarily rely on patient history and clinical presentation, the advancement of spike protein assays holds promise for more precise tracking and treatment of these conditions.

Although our review primarily focuses on mechanistic and clinical aspects, we recognize the increasing value of in silico toxicity prediction models for evaluating the potential adverse effects of spike protein variants and related vaccine components. Computational approaches such as molecular docking and molecular dynamics simulations have been used to assess the cytotoxicity and safety profiles of viral proteins, including the SARS-CoV-2 spike protein [343, 344]. Expanding and integrating these methods into future research could enhance our understanding of spike-related toxicities and support the rational design of safer vaccines and therapeutics.

Further research is needed to understand the factors influencing long-term spike protein persistence and to develop effective diagnostics and treatments tailored to individual patient profiles.

## Figures and Tables

**Table 1:** An overview of pathological mechanisms of spike protein.

Interacting protein	Interaction	Physiological impact	Clinical evidence
ACE-2	Binding of spike receptor binding domain (RBD) to ACE2 [98, 99]	Cell entry of SARS-CoV-2 (in the case of acute infection) [100], thrombosis [93], myocarditis/pericarditis [93], and vasculitis/endotheliitis [93]	Myocarditis [101], thrombosis/thrombocytopenia [102], vasculitis [103], and endotheliitis [104]
CD-147	Inhibition of CD-147 [105]	Myocarditis/pericarditits [106], microvascular disease [106], and hemolytic anemia [107]	Myocarditis [101], microvascular disease [108], and hemolytic anemia [109, 110]
Toll-like receptors	Binding/activation TLR4 [111], activation of TLR2 [112]	Cytokine release and inflammation [112–115]	Inflammatory disorders [116–118]
High-affinity estrogen receptor (ERα)	Binding/modulation to ERα [119]	Menstrual irregularities [120]	Menstrual irregularities [120, 121]
P53 BP1, BRCA1	Interaction with spike S2 subunit observed in silico [122]	Potential inhibition of tumor suppression mechanisms [123]	Non-Hodgkin lymphoma [124], myeloproliferative disorders [125]
Spike proteins or other potentially misfolding proteins	Prion-like propagation of spike aggregates [126]. Interaction with human prion protein and amyloid beta peptide [127]	Potential neurodegeneration [128] and blood microclots [126]	Dementia [129, 130]
Functional α7 nicotinic acetylcholine receptor (α7nAChR)	A helical motif in the neck of spike protein downregulates cell surface α7nAChR [131]	Neuropsychiatric symptoms [132]	Chronic fatigue [133], cognitive deficits [134]
Blood–brain barrier (BBB)	Degradation of barrier function [135, 136]. Relative permeability of BBB to spike [137, 138]	Possible contributor to neurologic manifestations [139]	Neurologic complications [140, 141]
Molecular mimicry	Several spike protein epitopes bear similarity to human proteins [142–144]	Varied symptomatology	Several disorders
Mitochondrial damage	Mechanism unknown, but relationship is established	Fatigue, brain fog	Chronic fatigue [133], cognitive deficits [134]

*Note:* Reproduced from [95] under a CC Attribution 4.0 international license (CC BY 4.0 DEED, https://creativecommons.org/licenses/by/4.0/).

**Table 2:** An overview of the spike protein-interacting proteins in the human body and their genetic variants, presenting a possible basis for variation in response to spike protein.

Interaction partner	Spike protein domain	Spike protein residues	Spike site variation	Interaction partner residues	SNPs	Potential interface inhibitors
ACE 2	RBD	Y449, Y453, L455, F456, Y473, F486, N487, Y489, Q493, G496, T500, N501, G502, Y505	F456L, F486V/P, Q493R, G496S, N501Y, Y505H	Q24, T27, D30, K31, H34, D38, Q42, L79, M82, Y83, E35, Y41, D355, K353, G354, R357, E374	I21V, E23K, K26R, T27A, N64K, T92I, Q102P, and H378R [157] R514G [158] rs2106806 and rs2106807 in combination [159] rs6629110 [159] K31R and E37K (lowered susceptibility) K26R and T92I (raised susceptibility) [160, 161] S19P, I21V, E23K, K26R, T27A, N64K, T92I, Q102P, and H378R (raised susceptibility) K31R, N33I, H34R, E35K, E37K, D38V, Y50F, N51S, M62V, K68E, F72V, Y83H, G326E, G352V, D355N, Q388L, and D509Y (lowered susceptibility) [161] V749V (rs35803318) and N690N (rs4646179) [162] K26R and S331F [163] R652 (interaction with TMPRSS2) [163] N720D (rs41303171) [164, 165] V749V (rs35803318), rs774469453, rs773676270, and rs2285666 [165] rs4646120 and rs2285666 [166] rs2074192 [167]	Nicotine (reduces ACE2 levels) [274] Nicotine [275] Investigational compounds [276]
CD147	RBD [172]	R403, K417, G446, Y449, Y453, L455, F456, P479, C480, N481, G482, V483, E484, G485, F486, C488, Y489, F490, L492, Q493, S494, Y495, G496, F497, Q498, P499, T500, N501, G502, V503, Y505, Q506	K417N/T, E484K/A, N501Y, Q493R, G496S, Q498R, Y505H, G446S, F456L, F486P, F490S, Q498R, N501Y, Y505H, F486V	54, 56, 57, 58, 59, 60, 61, 62, 74, 82, 83, 102, 106, 129, 130, 131, 132, 133, 135, 164, 165, 188, 190, 191, 192 Surrounding 37, 38, 63, 64, 65, 73, 75, 77, 78, 79, 80, 81, 84, 86, 92, 100, 103, 104, 105, 107, 108, 127, 128, 136, 137, 138, 162, 163, 166, 186, 189, 193, 194	rs8259 [174, 176] rs371073966 [175] E208K (rs104894669) rs2283574, rs6757, rs8637, rs4919862, rs6758, rs8259, rs4919859, and rs28915400 [176] Others [177]	Azithromycin (downregulator of CD-147) [171]. αCD147 (antibody) [277]
TLR1		ASN87, THR51, TYR204, SER305, THR761, THR240, and ASN30 ILE105, VAL36, PHE58, PHE220, and LEU117	No variation	SER399, HIS370, THR372, CYS368, and GLN402 MET397, ALA391, PHE350, and LEU377	rs5743551 [243]	Curcumin [278] Rationally designed compounds [279] Phloretin [280]
TLR2					R753Q (rs5743708) [244] rs3804100 [243]	Phloretin [280] Ortho-vanillin [281] N-Acetyl cysteine [282]
TLR4	Spike S1 [239]	S221, N280, T588, T208, N657, Y204, F562, L226, P289, I584	No variation in residues	N409, N333, S386, S352, H431, N361, L385, V411, F342, F408	rs4986790, rs4986791 [243, 244] D299G (rs4986790) [283] (n.s.) and T399I (rs4986791) [246] Candidate SNPs N409S, S386N, N361D/T/S/K, L385F, V411I, F342Y	N-Acetyl Cysteine [282] Reviewed in [284]
TLR6	S1	N536, T581, Q563, S221, Q564, and Y38 F559, L582, L552, F565, P561, and I587	No variation	H350, N423, N438, N387, H345, and T302 P349, L382, M335, I338, L304, and I430	S249P (rs5743810) [243] V327M (rs3796508) [285]	Baicalin [286]
ER-alpha	(NRC) LXD-like motif on the S2 subunit [119]	S816-L822, T859-L865	No variation	Helix 11. L404, E405, G406	None investigated in COVID-19 outcomes.	Investigational compounds [287], estrogen
P53	S2 [122]	D1165, L1166, N1173	No variation	T281, R270, R277, H175	No validated polymorphisms in proximity to interaction site	Investigational in silico [288]
BRCA1	S2	D1165, G1171, N1173	No variation	K1648, R1649, H1672	S1613G [289], M1652I [290],	Investigational peptides against S2 subunit of spike [291] Taxodione, Withaferin D [292].
BRCA2		D1165, L1166, S1175	No variation	E916, L938, R1117	Not a significant site of variation.	Taxodione, Camptothecin, Glaucarubinone [292]
α7nAChR	Helical domain in spike neck [131]	Y674-R685 [293]	N679K, P681H/R	W171, Y210, Y217, Y115 [294]	rs16969968 [295] Candidate SNPS: M1I, W9L, L60P, Y30C, R101H, C164R, L87S, D104E, I112V, S20F, A373P, Y233C, N381D, C241W, A434V, G424R, R363H, R446C, V395M, R448H	Nicotine, acetylcholine [296, 297]
TMPRSS2		Cleavage site R685, S686, R815, S816	No variation	H296, S441, S460	V160M (rs12329760) [158] 331G > A, c.23G > T, and c.589G > A [298] G385G (rs61735794) G290G (rs2298659) I256I (rs17854725) Y180Y (rs61735789) V160M (rs12329760) T75T (rs3787950) P63P (rs61735792) [162] rs75603675, rs12329760, and rs200291871 [164] V160M (rs12329760) [299] rs12329760, rs2070788, rs383510 [300] V197M (rs12329760) [301]	Reviewed in [302] Genistein, Quercetin [303]

*Note:* Variations in the interaction interface are shown for both the spike and human proteins, especially if clinically significant for COVID-19 infection. Highlighted residues are either variable or near a variable residue.

**Table 3:** Biomarkers that can be altered in the case of vaccine injury.

Biomarker	Upper limit of normal
Peak cardiac troponin (T)	14 ng/L [49]
Brain natriuretic Peptide (BNP)	35 ng/L [325]
N-terminal prohormone of brain natriuretic peptide (NT-proBNP)	125 ng/L (under 75 years of age) [325] 450 ng/L (over 75 years of age) [325]
C-reactive protein (CRP)	8 mg/L [49]
D-dimer	(patient's age in years × 10 mcg/L)^∗^ [326]

^∗^For patients 50 years or older.

**Table 4:** Basis of diagnostic difference between vaccinal and viral SARS-CoV-2 spike protein.

Vaccine spike	Viral spike
No N protein present [217]	N protein present [333]
Sequence identical to vaccine sequence [334]	Sequence much less constrained, reflects currently circulating variants [335]
Locked into prefusion conformation [336, 337]	Conformationally flexible [338]

## Data Availability

Data sharing is not applicable to this article as no new data were created or analyzed in this study.

## Supporting Information

Graphical Abstract. The factors affecting individual susceptibility to COVID-19 vaccine adverse effects. These are separated into factors related to the vaccine product itself, factors surrounding administration, host metabolic factors, host pharmacogenetics, and the pharmacogenomics of the spike protein.
