# Corrigendum to “The Possible Mechanistic Basis of Individual Susceptibility to Spike Protein Injury”

**DOI:** 10.1155/av/9806840

**Published:** 2025-09-16

M. Halma, P. Vottero, J. Thorp, T. Peers, J. Tuszynski, and P. Marik, “The Possible Mechanistic Basis of Individual Susceptibility to Spike Protein Injury,” *Advances in Virology* 2025 (2025): 7990876, https://doi.org/10.1155/av/7990876.

In the article titled “The Possible Mechanistic Basis of Individual Susceptibility to Spike Protein Injury,” there was an error in affiliation 3. The correct affiliation is shown below:

Department of Biomedical Engineering, University of Alberta, Alberta, Edmonton, Canada

We apologize for this error.

