# Supplementary material for: The Possible Mechanistic Basis of Individual Susceptibility to Spike Protein Injury
Source: Adv Virol. 2025 Jun 24;2025:7990876. doi: 10.1155/av/7990876 (PMC12213048; doi:10.1155/av/7990876)
Supplement: Supporting Information — Additional supporting information can be found online in the Supporting Information section. [file 7990876.f1.pdf]

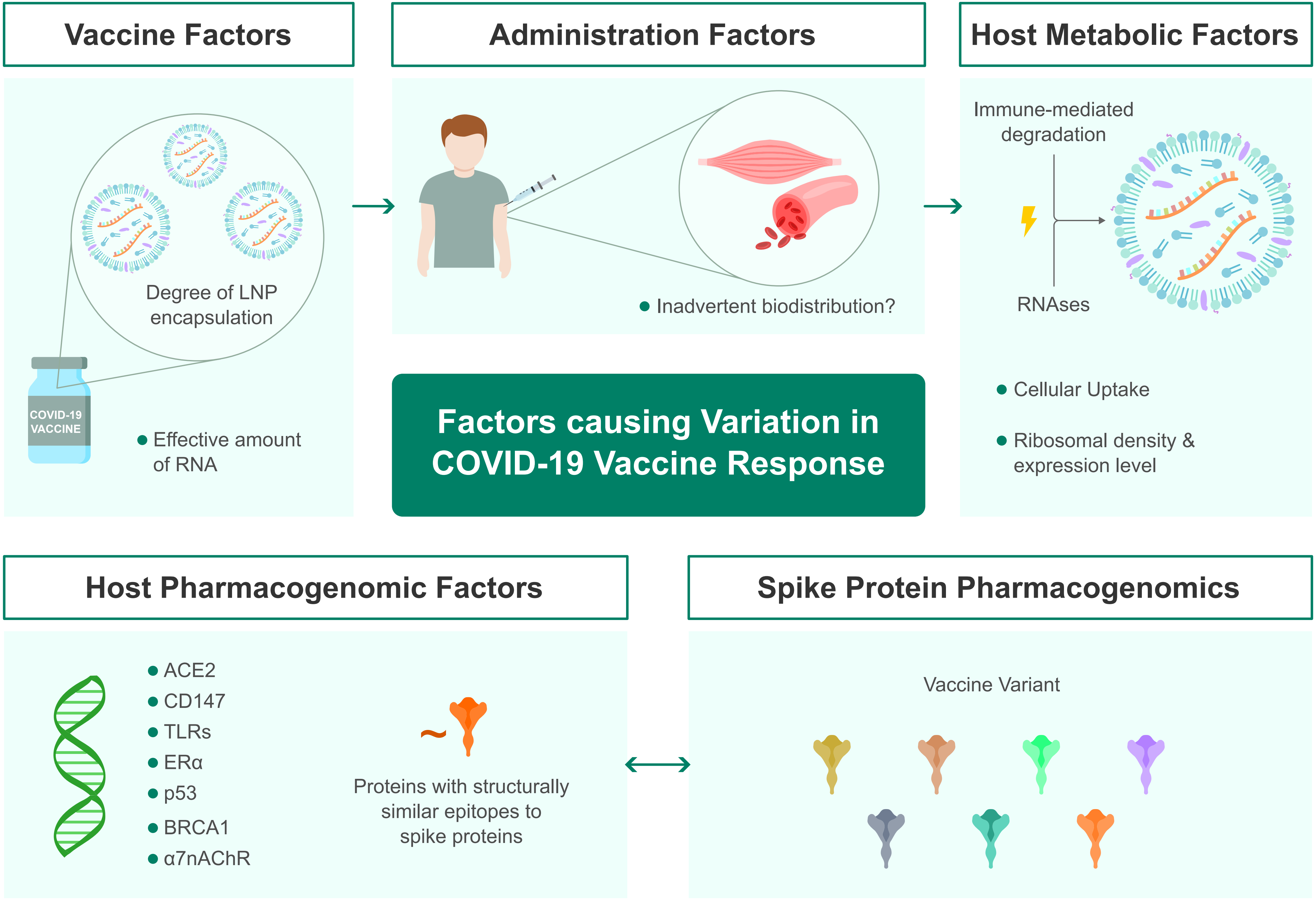

Vaccine Factors
Administration Factors
Host Metabolic Factors
Degree of LNP encapsulation
COVID-19 VACCINE
Effective amount of RNA
Inadvertent biodistribution?
Immune-mediated degradation
RNAses
Cellular Uptake
Ribosomal density & expression level
Factors causing Variation in COVID-19 Vaccine Response
Host Pharmacogenomic Factors
Spike Protein Pharmacogenomics
ACE2
CD147
TLRs
ERα
p53
BRCA1
α7nAChR
Proteins with structurally similar epitopes to spike proteins
Vaccine Variant
